# Supplementary material for: Survey of potentially inappropriate prescriptions for common cold symptoms in Japan: A cross-sectional study
Source: PLoS One. 2022 May 12;17(5):e0265874. doi: 10.1371/journal.pone.0265874 (PMC9098006; doi:10.1371/journal.pone.0265874)
Supplement: S2 Appendix — (DOCX) [file pone.0265874.s002.docx]

**Patient Information Sheet　　（1）date of response / /**

**【Inclusion criteria】**

**(2) Did you visit the doctor for one or more of the following symptoms: fever, headache, cough, nasal congestion,**

**sore throat.**

**□Yes（1）　□No（0）**

**(3)Are your symptoms similar to those you have had before?　　 　　　 □Yes（1）　□No（0）**

**(4)Age 　　　　　　　　　　　　　　　　　　 　years old**

**(5) Is this prescription medication for you to take?　　　　 　　　　□Yes（1）　□No（0）**

**(6)Are only drugs that relieve cold symptoms prescribed? 　　□Yes（1）　□No（0）**

**【Exclusion criteria】**

**(7) Do you have any underlying medical conditions?　　 　　 □Yes（1）　□No（0）**

**(8)** **Are you pregnant or breastfeeding?　　　　　　 　　　　　　　　 　□Yes（1）　□No（0）**

**(9) Is Japanese kanpo prescribed?　　　　　　　　　　　 □Yes（1）　□No（0）**

**(10) Are you taking any medications other than those that relieve cold symptoms on a regular basis?**

**□Yes（1）　□No（0）**

**# Remarks**

**【Patient consent】**

Thank you for your cooperation. The patient information sheet will be collected and may be used for research on prescription medications for common cold. No individual’s identity will be added in this questionnaire. In addition, patients will not be disadvantaged in any way by providing consent or by the content of the questionnaire.

Please cooperate with this research for the development of medical science and the reduction of medical costs in Japan.

**(11)□I agree to the use of my survey information.（1）　 □I don't agree with it.（0）**

*Yasuhisa Nakano ,Shimane University Faculty of Medicine*

*Takashi Watari , General Medicine Center, Shimane University*

*Yuji Takaki, Midori Pharmacy Co. Ltd.*

**【Details of interviewees】**

**(12) Gender** 　　　　　 **□Male（1）　□Female（0）**

**(13)** **Did your doctor explain it as "cold, common cold, upper respiratory tract infection, pharyngitis, etc."?**

**□Yes（1）　□No（0）（contents）**

**◇What are your symptoms today?**

| Patient's symptoms | ◽️（Please check.✔︎） |
| --- | --- |
| (14) Runny nose | **□Yes（1）　□No（0）** |
| (15) Nasal congestion | **□Yes（1）　□No（0）** |
| (16) Sneezing | **□Yes（1）　□No（0）** |
| (17) Cough | **□Yes（1）　□No（0）** |
| (18) Phlegm | **□Yes（1）　□No（0）** |
| (19) Fever (If your answer is "Yes", please indicate the temperature) | **□Yes（1）→（　　　℃）**  **□No（0）** |
| (20) Headache | **□Yes（1）　□No（0）** |
| (21) Fatigue | **□Yes（1）　□No（0）** |
| (22) Abdominal pain | **□Yes（1）　□No（0）** |
| (23) Sore throat、dysphagia | **□Both（2）　□Only Sore throat(1) 　□None（0）** |
| (24) Others | **□Yes（1）　□No（0）** |

**(25)　How many days have the above symptoms been present?　　　　approx.　　　　days**

**(26)** **Do you have any allergies?　 　 □Yes（1）（contents　　　　　　 ）　□No（0）**

**(27)**  **Do you smoke?　□Yes（1）→ ( 　 cigarettes a day for 　 years # Packs Year )**

**□No（0）**

**【Details of medication】 　　date / /**

| **Medication** | **Effect or mechanism** | **Prescription** | | **Standard** | **Number of days and frequency of medication**  **Dosage** |
| --- | --- | --- | --- | --- | --- |
| PL combination garanukes | **Multi-ingredient cold medication** | **□Yes（1）　□No（0）** | |  |  |
| SG combination garanukes | Antifebrile, analgesic, and anti-inflammatory drug | **□Yes（1）　□No（0）** | |  |  |
| Carbocisteine | Expectorant drug | **□Yes（1）　□No（0）** | |  |  |
| Bromhexine | Expectorant drug | **□Yes（1）　□No（0）** | |  |  |
| Ambroxol | Expectorant drug | **□Yes（1）　□No（0）** | |  |  |
| Chlorpheniramine | H1 receptor antagonist(First-generation) | **□Yes（1）　□No（0）** | |  |  |
| Loratadine  Levocetirizine  Nipolazine  Oxatomide  Bepotastine | H1 receptor antagonist( Second-generation) | **□Yes（1）　□No（0）** | |  |  |
| Pranlukast  Montelukast | Leukotoluene receptor antagonist | **□Yes（1）　□No（0）** | |  |  |
| Codeine phosphate | narcotic Antitussives | **□Yes（1）　□No（0）** | |  |  |
| Dextromethorphan | non-narcotic antitussives | **□Yes（1）　□No（0）** | |  |  |
| Dimemorfan | non-narcotic antitussives | **□Yes（1）　□No（0）** | |  |  |
| Chipepidine hibenzate | non-narcotic antitussives | **□Yes（1）　□No（0）** | |  |  |
| Mometasone | Steroid | **□Yes（1）　□No（0）** | |  |  |
| **Prednisolone** | Steroid | **□Yes（1）　□No（0）** | |  |  |
| Ipratropium | anticholinergic agent | **□Yes（1）　□No（0）** | |  |  |
| α１agonist | | **□Yes（1）　□No（0）** | |  |  |
| Decalinium chloride (Cough drop) | Pharyngitis | **□Yes（1）　□No（0）** | |  |  |
| Tranexamic acid | Pharyngitis | **□Yes（1）　□No（0）** | |  |  |
| Povidone‐iodine, sodium azulene sulfonate (mouthwash) | Pharyngitis | **□Yes（1）　□No（0）** | |  |  |
| Tulobuterol  Terbutaline | β2 stimulants | **□Yes（1）　□No（0）** | |  |  |
| Theophylline | Phosphodiesterase inhibitor | **□Yes（1）　□No（0）** | |  |  |
| Diclofenac sodium  Loxoprofen  Thiaramide | NSAIDｓ | **□Yes（1）　□No（0）** | |  |  |
| Acetaminophen | Analgesia | **□Yes（1）　□No（0）** | |  |  |
| Amoxicillin | Penicillin antibiotics | **□Yes（1）　□No（0）** | |  |  |
| Cephkapen | Cephem antibiotics | **□Yes（1）　□No（0）** | |  |  |
| Cefdinir | Cephem antibiotics | **□Yes（1）　□No（0）** | |  |  |
| Cefmenoxime （Nose drops） | Cephem antibiotics | **□Yes（1）　□No（0）** | |  |  |
| Azithromycin | Macrolide antibiotics | **□Yes（1）　□No（0）** | |  |  |
| Clarithromycin | Macrolide antibiotic | **□Yes（1）　□No（0）** | |  |  |
| Galenoxacin | New quinolone antibiotic | **□Yes（1）　□No（0）** | |  |  |
| Levofloxacin | New quinolone antibiotic | **□Yes（1）　□No（0）** | |  |  |
| Lactobacillus bifidus | Internal disorders | **□Yes（1）　□No（0）** | |  |  |
| Lactic acid bacilli | Internal disorders | **□Yes（1）　□No（0）** | |  |  |
| Lactomin | Internal disorders | **□Yes（1）　□No（0）** | |  |  |
| Domperidone | Gastrointestinal symptoms | | **□Yes（1）　□No（0）** |  |  |
| Marslen*  Amount of ingredients in 1 g  azulene sodium sulfonate 3 mg  L‐glutamine 990 mg | Gastritis | | **□Yes（1）　□No（0）** |  |  |
| Loperamide | Diarrhea | | **□Yes（1）　□No（0）** |  |  |
| Tramazoline | Nasal inflammation | | **□Yes（1）　□No（0）** |  |  |
| **Drug cost** | **yen** | | | | |

**Pharmacist's judgment-i**

|  | Item | comment |
| --- | --- | --- |
| (28)  Pharmacist's name | - Takaki (1) - Adachi (2) - Nakano (3) - Watanabe (4) |  |
| (29)  Evidence for prescription drugs | □enough （1）  □possibility of insufficiency（0） |  |
| (30)  Are the symptomatic medications appropriate for the patient's symptoms? | □appropriate(1)  □potentially inappropriate(0) |  |
| (31)  Quantity of medicines | □appropriate(1)  □potentially inappropriate(0) |  |
| (32)  Contraindications to medicines | □Yes(1)  □No（2） |  |
| (33)  Overall judgment | □appropriate prescription（1）  □potentially inappropriate prescription（0） | |

**Pharmacist's judgment-ii**

|  | Item | comment |
| --- | --- | --- |
| (34)  Pharmacist's name | - Takaki (1) - Adachi (2) - Nakano (3) - Watanabe (4) |  |
| (35)  Evidence for prescription drugs | □enough （1）  □possibility of insufficiency（0） |  |
| (36)  Are the symptomatic medications appropriate for the patient's symptoms? | □appropriate(1)  □potentially inappropriate(0) |  |
| (37)  Quantity of medicines | □appropriate(1)  □potentially inappropriate(0) |  |
| (38)  Contraindications to medicines | □Yes(1)  □No（2） |  |
| (39)  Overall judgment | □appropriate prescription（1）  □potentially inappropriate prescription（0） | |

**Comprehensive judgment of the physician and the pharmacist**

□appropriate prescription（1）

□potentially inappropriate prescription（0）
