# Supplementary material for: Survey of potentially inappropriate prescriptions for common cold symptoms in Japan: A cross-sectional study
Source: PLoS One. 2022 May 12;17(5):e0265874. doi: 10.1371/journal.pone.0265874 (PMC9098006; doi:10.1371/journal.pone.0265874)
Supplement: S3 Appendix — (PPTX) [file pone.0265874.s003.pptx]

## Slide 1
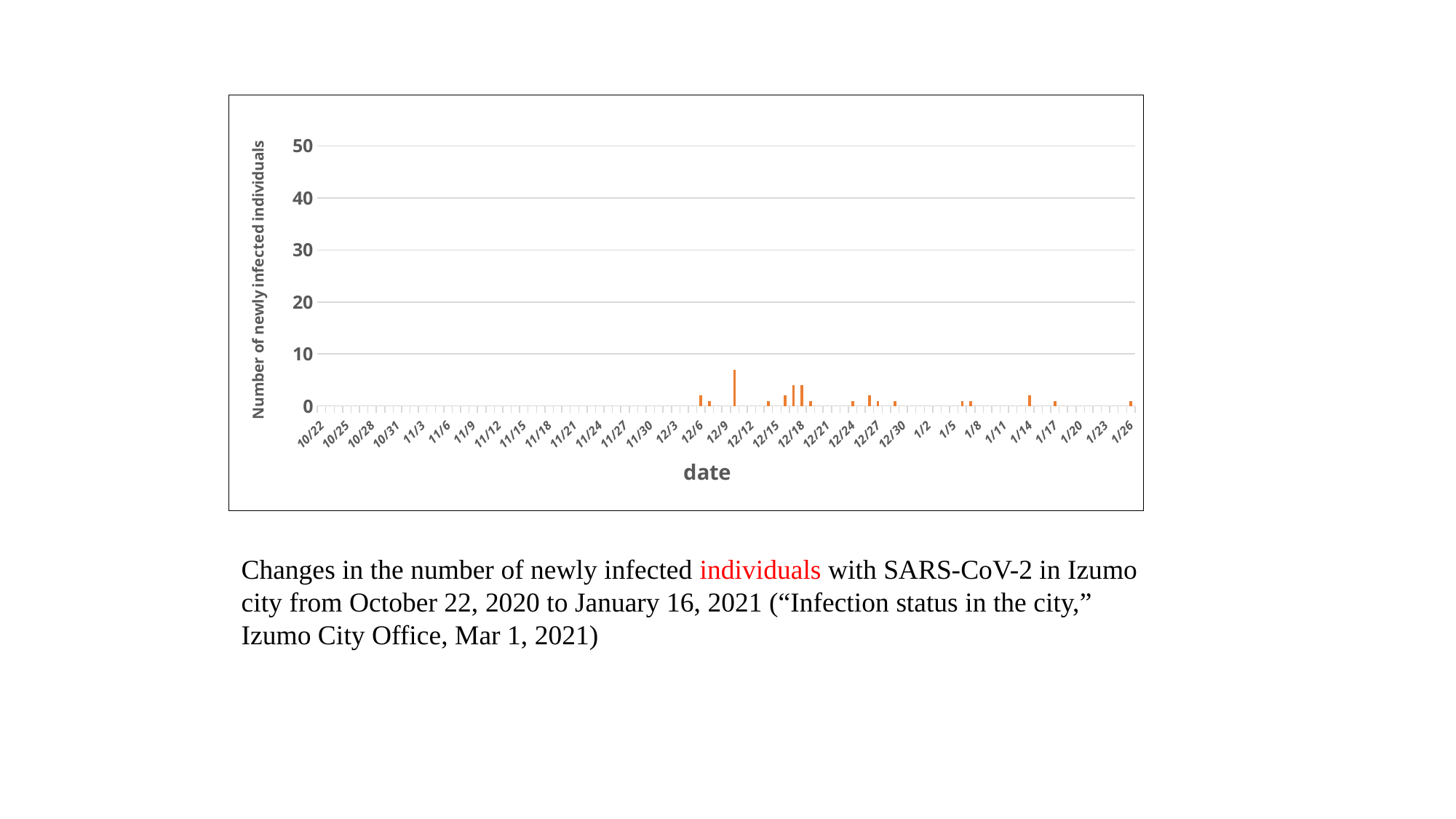

### Chart
| Category | |
|---|---|
| 44126 | 0.0 |
| 44127 | 0.0 |
| 44128 | 0.0 |
| 44129 | 0.0 |
| 44130 | 0.0 |
| 44131 | 0.0 |
| 44132 | 0.0 |
| 44133 | 0.0 |
| 44134 | 0.0 |
| 44135 | 0.0 |
| 44501 | 0.0 |
| 44502 | 0.0 |
| 44503 | 0.0 |
| 44504 | 0.0 |
| 44505 | 0.0 |
| 44506 | 0.0 |
| 44507 | 0.0 |
| 44508 | 0.0 |
| 44509 | 0.0 |
| 44510 | 0.0 |
| 44511 | 0.0 |
| 44512 | 0.0 |
| 44513 | 0.0 |
| 44514 | 0.0 |
| 44515 | 0.0 |
| 44516 | 0.0 |
| 44517 | 0.0 |
| 44518 | 0.0 |
| 44519 | 0.0 |
| 44520 | 0.0 |
| 44521 | 0.0 |
| 44522 | 0.0 |
| 44523 | 0.0 |
| 44524 | 0.0 |
| 44525 | 0.0 |
| 44526 | 0.0 |
| 44527 | 0.0 |
| 44528 | 0.0 |
| 44529 | 0.0 |
| 44530 | 0.0 |
| 44166 | 0.0 |
| 44167 | 0.0 |
| 44168 | 0.0 |
| 44169 | 0.0 |
| 44170 | 0.0 |
| 44171 | 2.0 |
| 44172 | 1.0 |
| 44173 | 0.0 |
| 44174 | 0.0 |
| 44175 | 7.0 |
| 44176 | 0.0 |
| 44177 | 0.0 |
| 44178 | 0.0 |
| 44179 | 1.0 |
| 44180 | 0.0 |
| 44181 | 2.0 |
| 44182 | 4.0 |
| 44183 | 4.0 |
| 44184 | 1.0 |
| 44185 | 0.0 |
| 44186 | 0.0 |
| 44187 | 0.0 |
| 44188 | 0.0 |
| 44189 | 1.0 |
| 44190 | 0.0 |
| 44191 | 2.0 |
| 44192 | 1.0 |
| 44193 | 0.0 |
| 44194 | 1.0 |
| 44195 | 0.0 |
| 44196 | 0.0 |
| 44197 | 0.0 |
| 44198 | 0.0 |
| 44199 | 0.0 |
| 44200 | 0.0 |
| 44201 | 0.0 |
| 44202 | 1.0 |
| 44203 | 1.0 |
| 44204 | 0.0 |
| 44205 | 0.0 |
| 44206 | 0.0 |
| 44207 | 0.0 |
| 44208 | 0.0 |
| 44209 | 0.0 |
| 44210 | 2.0 |
| 44211 | 0.0 |
| 44212 | 0.0 |
| 44213 | 1.0 |
| 44214 | 0.0 |
| 44215 | 0.0 |
| 44216 | 0.0 |
| 44217 | 0.0 |
| 44218 | 0.0 |
| 44219 | 0.0 |
| 44220 | 0.0 |
| 44221 | 0.0 |
| 44222 | 1.0 |Changes in the number of newly infected individuals with SARS-CoV-2 in Izumo city from October 22, 2020 to January 16, 2021 (“Infection status in the city,” Izumo City Office, Mar 1, 2021)
