# Supplementary material for: Survey of potentially inappropriate prescriptions for common cold symptoms in Japan: A cross-sectional study
Source: PLoS One. 2022 May 12;17(5):e0265874. doi: 10.1371/journal.pone.0265874 (PMC9098006; doi:10.1371/journal.pone.0265874)
Supplement: Attachment — Submitted filename: Reviewers Comments.docx [file pone.0265874.s004.docx]

Reviewer’s comments

Inappropriate use of antibiotics has often been pointed out in Japan. The study was first prospective observational study which showed that 90% of prescriptions including antibiotics for common cold symptoms was inappropriate. This is an interesting document by depth interviews with patients who had been prescribed medication to relieve cold symptoms.

This paper will be considered acceptable if the following comments are properly addressed.

1. Method

I would suggest adding more in the Statistical analyses section on the statistical methods used for deriving results described in the results section (including ones included in the Tables). For example, currently there is no mention on IQRs in the statistical analyses section – please add an explanation on this.

2. Results

How many physicians have prescribed the drugs in this time? What are the specialties of the doctors who prescribed it? What percentage of prescribing physicians belong to a hospital or clinic? Please add their characteristics if possible.

3. Discussion
1) Four issues on the part of physicians have been pointed out as factors in inappropriate prescribing (Cephem antibacterials, β2-stimulants, and new quinolone antibacterials). What about the role of pharmacists, such as their intervention against inappropriate prescriptions? For example, in Japan, as a countermeasure against polypharmacy, a pharmaceutical management fee has been newly established as a dispensing fee, and pharmacists are encouraged to propose drug reduction.

Since there is no pharmacist's view for inappropriate prescribing in this document, please add their perspective.

2) It has been shown that medication costs are higher in the inappropriate prescription

group, but this point was not good enough explained, though there are some words about Japanese healthcare system in the “Discussion”

3) It was described the first factor is that patients may be seeking medications from doctors in this document. However, some authors interviewed the patients actually. Did they confirm this factor? What was the patients' view?

From the perspective of “Choosing Wisely”, how do the authors think about the health literacy education for patients?
