# Supplementary material for: Survey of potentially inappropriate prescriptions for common cold symptoms in Japan: A cross-sectional study
Source: PLoS One. 2022 May 12;17(5):e0265874. doi: 10.1371/journal.pone.0265874 (PMC9098006; doi:10.1371/journal.pone.0265874)
Supplement: Attachment — Submitted filename: Reviewers comments 2_2022.2.10.docx [file pone.0265874.s006.docx]

Reviewer
1. Method
I would suggest adding more in the Statistical analyses section on the statistical methods used for deriving results described in the results section (including ones included in the Tables). For example, currently there is no mention on IQRs in the statistical analyses section – please add an explanation on this.

Response:
Changes (Method_Statistical Analyses)
P7, L148

Dear author

Your comment has been addressed.

Reviewer
2. Results
How many physicians have prescribed the drugs in this time? What are the specialties of the doctors who prescribed it? What percentage of prescribing physicians belong to a hospital or clinic? Please add their characteristics if possible.
Response

Addition (Discussion_ Limitation)
P18, L347 to P19, L359 (marked_manuscript)

Dear author

Your comment has been addressed, but please make it more briefly and more compact.

Reviewer
3.Discussion
1) Four issues on the part of physicians have been pointed out as factors in inappropriate prescribing (Cephem antibacterials, β2-stimulants, and new quinolone antibacterials). What about the role of pharmacists, such as their intervention against inappropriate prescriptions? For example, in Japan, as a countermeasure against polypharmacy, a pharmaceutical management fee has been newly established as a dispensing fee, and pharmacists are encouraged to propose drug reduction.
Since there is no pharmacist's view for inappropriate prescribing in this document, please add their perspective.

Response:

Addition (Discussion_ Pharmacists' views on potentially inappropriate prescription)
P17, L306

Dear author

I understand it. However, I think the following point is one of the major problem faced by pharmacists.　This is because pharmacists in Japan have not yet established the professional competence to review and suggest prescriptions for pharmacotherapy.

Reviewer
3.Discussion
2) It has been shown that medication costs are higher in the inappropriate prescription
group, but this point was not good enough explained, though there are some words about Japanese healthcare system in the “Discussion”

Response
n_ Considerations for drug costs)
P17, L322 (marked_manuscript)

Dear author

I don't think the following explanation adequately describes the actual situation in Japan.　"Japan's universal health insurance system covers all prescription drugs without restrictions.”

For example, there is the Diagnosis Procedure Combination (DPC), and there is a system for polypharmacy, which provides reimbursement for the dispensing of drugs by reducing the number of drugs. Therefore, how about "Japan's universal health insurance system covers all prescription drugs generally.” ?

Reviewer
3.Discussion
3)It was described the first factor is that patients may be seeking medications from doctors in this document. However, some authors interviewed the patients actually. Did they confirm this factor? What was the patients' view?
From the perspective of “Choosing Wisely”, how do the authors think about the health literacy education for patients?

Response

Dear author

How about the following references as citations of Choosing Wisely?

- Consumer Reports

<https://www.consumerreports.org/doctors/questions-to-ask-your-doctor/>

or

- Muscat DM, et al. Evaluation of the Choosing Wisely Australia 5 Questions resource and a shared decision-making preparation video: protocol for an online experiment. BMJ Open 2019;9:e033126

<https://bmjopen.bmj.com/content/9/11/e033126>

Dear author

Finally, after reading the submitted paper more precisely again, I would like to mention an additional point.

The design of this study is a cross sectional study, and thus, it seems inappropriate to refer to this study as a prospective observational study. The reason is as follows; it is cross sectional because they looked at the prevalence of whether or not there had been an inappropriate prescription, where the survey lasted for 3 months – but in fact, the study participants were not followed for 3 months. It is recommended that the authors consult an epidemiologist.
