# Supplementary material for: Survey of potentially inappropriate prescriptions for common cold symptoms in Japan: A cross-sectional study
Source: PLoS One. 2022 May 12;17(5):e0265874. doi: 10.1371/journal.pone.0265874 (PMC9098006; doi:10.1371/journal.pone.0265874)
Supplement: Attachment — Submitted filename: response_to_reviewer_.docx [file pone.0265874.s007.docx]

March 4, 2022

Dear *PLOS ONE* Editor:

Thank you for giving us the opportunity to revise our manuscript. The constructive suggestions and feedback provided by the Reviewer have enabled us to substantially improve the quality of our paper. We have provided a point-by-point response to the Reviewer’s suggestions. The revised portions have been indicated in green highlight in the revised manuscript.

Once again, thank you for your thorough and supportive peer review.

On behalf of all the authors,

Yours sincerely,

Corresponding author

Takashi Watari, MD, MHQS, MCTM, Ph.D

Shimane University Hospital, General Medicine Center, Shimane, Japan

89-1, Enya-cho, Izumo shi, Shimane, 693-8501, Japan

e-mail: [wataritari@gmail.com](mailto:wataritari@gmail.com); Phone: +81-853-20-2005; Fax: +81-853-20-2375

**Reviewer**

***2. Results***

*How many physicians have prescribed the drugs in this time? What are the specialties of the doctors who prescribed it? What percentage of prescribing physicians belong to a hospital or clinic? Please add their characteristics if possible.*

***Response***

*Addition (Discussion_ Limitation)*

*P18, L347 to P19, L359 (marked_manuscript)*

***Dear author***

*Your comment has been addressed, but please make it more briefly and more compact.*

**Response:**

We appreciate and agree with the reviewers’ comment. The following changes have been made in the revised manuscript:

**Change (Discussion_ Limitation)**

**Page 19, Lines 362 to 367 (marked_manuscript)**

**Limitation:** Sixth, the details regarding the prescribing physician were not investigated in this study as the Ethics Committee stipulates that "the content that identifies the prescribing physician (specialty, affiliation, and the number of individuals) must not be added to the content of the interview because it may cause disadvantages to the prescribing physician.” We believe that the prescribing physician's details are important factors and further research is needed.

**Reviewer**

***Reviewer***

*3.Discussion*

*2) It has been shown that medication costs are higher in the inappropriate prescription*

*group, but this point was not good enough explained, though there are some words about Japanese healthcare system in the “Discussion”*

***Response***

*Discussion_ Considerations for drug costs)*

*P17, L322 (marked_manuscript)*

***Dear author***

*I don't think the following explanation adequately describes the actual situation in Japan.　"Japan's universal health insurance system covers all prescription drugs without restrictions.”*

*For example, there is the Diagnosis Procedure Combination (DPC), and there is a system for polypharmacy, which provides reimbursement for the dispensing of drugs by reducing the number of drugs.* *Therefore, how about "Japan's universal health insurance system covers all prescription drugs generally.” ?*

**Response**

We appreciate and agree with the reviewers’ comment. The following changes have been made in the revised manuscript:

**Addition (Discussion_** **Considerations for drug costs)**

**Page 18, Line 331 (marked_manuscript)**

**Considerations for drug costs:** Japan's universal health insurance system covers all prescription drugs generally.

**Reviewer**

***Reviewer***

*3.Discussion*

*3)It was described the first factor is that patients may be seeking medications from doctors in this document. However, some authors interviewed the patients actually. Did they confirm this factor? What was the patients' view?*

*From the perspective of “Choosing Wisely”, how do the authors think about the health literacy education for patients?*

*Response*

***Dear author***

*How about the following references as citations of Choosing Wisely?*

* Consumer Reports*

*https://www.consumerreports.org/doctors/questions-to-ask-your-doctor/*

*or*

** *Muscat DM, et al. Evaluation of the Choosing Wisely Australia 5 Questions resource and a shared decision-making preparation video: protocol for an online experiment. BMJ Open 2019;9:e033126*

*https://bmjopen.bmj.com/content/9/11/e033126*

**Response**

We appreciate and agree with the reviewers’ comment. The following changes have been made in the revised manuscript:

**Addition (Discussion_ Considerations for drug costs)**

**Page 18, Line 334 to 341 (marked_manuscript)**

**Considerations for drug costs:** As part of the Choosing Wisely campaign, it is generally recommended that patients ask their doctors the following five questions [46]. (1) Do I really need this test or procedure? (2) What are the risks? (3) Are there simpler, safer options? (4) What happens if I do not do anything? (5) What are the costs? Patients must be aware of their own "treatment" by referring to the above questions to promote the appropriate use of medicines in Japan. It is necessary to incorporate education and measures that consider the interactive opinions of the medical professionals and patients.

**Reviewer**

*Finally, after reading the submitted paper more precisely again, I would like to mention an additional point.*

*The design of this study is a cross sectional study, and thus, it seems inappropriate to refer to this study as a prospective observational study. The reason is as follows; it is cross sectional because they looked at the prevalence of whether or not there had been an inappropriate prescription, where the survey lasted for 3 months – but in fact, the study participants were not followed for 3 months. It is recommended that the authors consult an epidemiologist.*

**Response**

We appreciate and agree with the reviewers’ comment. The following changes have been made in the revised manuscript:

**Changes (marked_manuscript)**

**Page 1, Line 2:** A Cross-Sectional Study

**Page 1, Line 28:** cross-sectional study

**Page 4, Line 82:** cross-sectional study

**Page 14-15, Line 251-252:** cross-sectional study
